# A Systematic Review of Mathematical Models of Dengue Transmission and Vector Control: 2010–2020

**DOI:** 10.3390/v15010254

**Published:** 2023-01-16

**Authors:** Samson T. Ogunlade, Michael T. Meehan, Adeshina I. Adekunle, Emma S. McBryde

**Affiliations:** 1 Australian Institute of Tropical Health and Medicine, James Cook University, Townsville 4811, Australia; 2 College of Medicine and Dentistry, James Cook University, Townsville 4811, Australia; 3 Defence Science and Technology Group, Department of Defence, Melbourne 3207, Australia

**Keywords:** dengue, transmission models, vector control, review

## Abstract

Vector control methods are considered effective in averting dengue transmission. However, several factors may modify their impact. Of these controls, chemical methods, in the long run, may increase mosquitoes’ resistance to chemicides, thereby decreasing control efficacy. The biological methods, which may be self-sustaining and very effective, could be hampered by seasonality or heatwaves (resulting in, e.g., loss of *Wolbachia* infection). The environmental methods that could be more effective than the chemical methods are under-investigated. In this study, a systematic review is conducted to explore the present understanding of the effectiveness of vector control approaches via dengue transmission models.

## 1. Introduction

Dengue is one of the world’s most threatening and widespread mosquito-borne diseases [1,2]. In recent decades, dengue has accounted for approximately 390 million new infections each year, with 96 million of these being symptomatic [3,4]. Most of the new annual infected cases (approximately 70% of 390 million) are distributed across Asia, while Africa, the Americas, and Oceania shared infection distributions of approximately 16.4%, 13.8%, and 0.2%, respectively [3]. The main vectors responsible for dengue transmission are *Aedes aegypti* and *Aedes albopictus* [5]. Dengue virus (DENV) has four distinct but closely related serotypes of the genus *Flavivirus*, namely: DENV-1; DENV-2; DENV-3; and DENV-4. When one recovers from one of these serotypes, it may provide lifelong immunity against that serotype. However, the cross-reactive immunity of the other serotypes is only temporary and partial [6]. Therefore, the subsequent infection of different serotypes of the dengue virus poses an increase in the risk of severe dengue viral infection [6]. The clinical manifestation includes headache, arthralgia, sudden high-grade fever, eye pain, nausea, and muscle ache [7]. Currently, there is no specific treatment for dengue. The efficacy of the vaccine that targets young patients depends on prior immunity to dengue, and it provides heterogeneous protection against the different serotypes [8,9]. The extent and severity of the burden imposed by dengue infection and disease have renewed calls for immediate intervention and control [10,11,12,13].

Vector control remains the most widely adopted technique to suppress the transmission of dengue [4,14]. This is because reducing the prevalence of dengue-carrying mosquitoes or inhibiting their transmission capacities typically poses a negligible risk of environmental contamination and demands little provision to sustain control [4,15].

There are three main approaches to vector control, namely, the chemical, environmental, and biological approaches [4,16]. Chemical methods involve the direct killing of mosquito vectors either by insecticide via indoor-residual spraying (IRS) or by limiting the reproduction of the vector population by chemically destroying mosquito breeding sites [4,17,18]. Environmental methods include emptying or covering water-filled containers, installing adequate water supply pipes, implementing efficient waste management strategies, and ensuring a clean environment [4]. Biological methods rely on the introduction of biological control agents, such as lavivorous fish, copepods, genetically modified mosquitoes, and *Wolbachia*-infected mosquitoes, which are incapable of transmitting arboviral pathogens [19,20,21]. Of these biological control methods, the *Wolbachia*-based strategy is becoming increasingly popular for controlling viral diseases (such as dengue) because it is potentially self-sustaining [15,22,23,24]. Although there is an emerging control method, mechanical control, that involves the mass trapping of mosquitoes using lethal traps and has been successful in controlling vectors transmitting dengue [25,26,27], mathematical models describing this method are scarce [28].

In practice, vector control—which addresses the suppression of the vector population and disruption of the viral transmission capabilities of mosquitoes—is the primary method for reducing dengue viral transmission. Unfortunately, these methods typically require considerable labour and monetary investments to achieve successful and sustained control and may also pose environmental risks (e.g., through the use of chemicides) [4,16]. The authors in [29] reviewed published articles on arboviral infections (such as dengue, Zika, and chikungunya) and their vector (*Aedes* mosquitoes) controls in general. They further assessed *Wolbachia*-based control studies for mitigating or eliminating arboviral infections and discussed gaps, such as the combination of the three (biological, chemical, and environmental) vector control methods and the use of two different *Wolbachia* strains that could be instrumental in developing models to estimate the impact of the controls. In this study, we examine the role of mathematical models in controlling the transmission of dengue and explore the present understanding of the effectiveness of vector controls in the last decade. This requires an extensive systematic search of the literature using field-related search terms in three different databases.

The introduction of mathematical models to understand viral infection dynamics has long been helpful in the area of disease control [30,31,32,33,34,35,36]. Several models involving vector control of the transmission of different dengue serotypes have been formulated and analysed [30,32,33,34,35,36,37,38,39,40,41,42,43,44,45,46,47,48,49,50,51,52,53,54,55,56,57,58,59,60,61,62,63,64,65]. Some studies [35,36,38,41,42,44,52] investigated dengue transmission models capturing the different dengue serotypes together with their vectors. Further, they described the dengue models by either host-vector transmission dynamics or purely by interactions between vectors. Here we review mathematical models of dengue vector control and identify literature modelling gaps in the last decade (from 2010 to 2020). We limited the time range to the last decade because some vector control techniques, such as *Wolbachia*-based techniques, were only recently successfully introduced [24,66], and a systematic review of dengue transmission models which accounts for vector control has been described up to early 2012 [36]. Another similar study by Perkins et al. [67] reviewed the dengue transmission models that covered a 40-year period (i.e., from 1970–2010). They used a standardized questionnaire to describe the various biological assumptions (corresponding to the Ross-Macdonald model assumptions) guiding each model and then gave both qualitative and quantitative findings. We carefully appraise published research articles describing the dengue transmission models and specifically classify these models according to the vector control method studied within a decade (i.e., from 2010–2020). This, in turn, will help reveal the literature gaps that will inform the development and modification of dengue models to account for effective vector control techniques.

## 2. Methods

The Preferred Reporting Items for Systematic Reviews and Meta-Analyses (PRISMA) guidelines [68] were used to conduct a systematic literature search. This search was performed using the MEDLINE, Web of Science (WOS), and SCOPUS databases from March to December 2020. We systematically used various keywords and/or synonyms, such as “dengue” OR “arbovirus”, together with “model” AND “control” OR “strategy” OR “technique” (see Additional File S1). Other keywords, such as “flavivirus”, “dengue virus”, “insect control”, and “communicable disease control”, were used to expand the search terms as some of the terms have been used interchangeably in the pool of literature. This review is aimed at the mathematical modelling of vector control methods in dengue transmission models. For each vector control method, we identify the underlying structure of the mathematical model, parameter assumptions, thresholds of implementations, and limitations.

### 2.1. Selection Criteria

At the initial stage of the search process, there were no restrictions for the time frame of the selected articles from each database used. However, it was later limited to 2010–2020. This is because (i) biological vector control techniques, in particular, *Wolbachia*-based control, were not significantly discussed until the last decade when the successful establishment of *Wolbachia* infection and its ability to block viral transmission in *Aedes* mosquitoes were reported [24,66], and (ii) another systematic review of the structure of dengue epidemiological transmission models, which includes vector control strategies, was conducted up to March 2012 [36]. 

The titles and abstracts of the articles irrelevant to the scope of the study were excluded from the articles of discussion (see details in the Section 3). Study articles published in non-English languages were removed from the considered pool of articles. Other referencing types, such as conference proceedings, serials, books, and book sections, were also removed. The inclusion criteria for these articles include the following:

- A representation of vectors or vector-host dynamics to control dengue transmission.
- A deterministic (DM), stochastic (SM), or network (NM) modelling approach using systems of ODEs.
- A vector control strategy leading to dengue viral reduction or elimination.

### 2.2. Data Extraction

**Figure 1:**
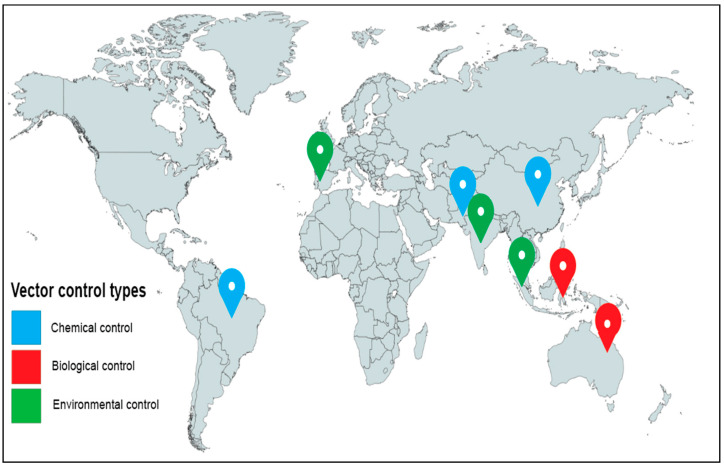
The distribution of the locations of the selected experimental studies used for validating models describing the vector control types.

The selected articles for this study were evaluated according to the modelling characteristics in terms of contributions made. These studies’ features include the aim and objectives, modelling methods, study location, vector control types, and key findings. It is important to mention that the vector control effectiveness of these studies was extracted from the findings and conclusions (Table 1). This is because the conclusive findings described in the studies provide a means for comparison and inference based on study effectiveness. Models were also categorised based on the year, vector control types, methods, and location of study to capture the general trend and geographical clusters of these models (Table 1). Figure 1 shows the distribution of the selected articles, stratified using the vector control types and location in which the controls were conducted. In other words, this shows the geographic clustering for where the experimental data were obtained for validating the vector control model outputs (Figure 1).

### 2.3. Assessment of Study Quality

We adapted and built upon the tool for the Assessment of Modelling Studies (AMS) [69], which is used for assessing modelling work in health and economics. In particular, we built upon the AMS by adding two more criteria (modelling methods and reporting conflicts of interest) to the ten existing criteria in the AMS (Table 2). The newly modified ASM tool used the quality assessment value formats in [70]. The tool comprises 12 criteria, and these criteria describe the characteristics of each of the articles selected for this review. Each criterion in the adapted tool used was assigned a rating from 0 to 2, where the following values represent the AMS criteria: 0 → absent, 1→ partially present, and 2 → fully present. Herein, the highest score for the modelling studies is 24 points. To compute the score y (in percentage) for an article, we used $y=\left(\frac{x}{24}\times 100\right)\%,\;$ where x is the number of points estimated for an article. Below, the quality of the articles is stratified into four categories: low (y≤ 50%), medium (50% < *y* ≤ 65%), high (65% < *y* ≤ 80%), and very high (*y* > 80%) (Table 2).

## 3. Results

### 3.1. Search Strategies and General Study Characteristics

**Figure 2:**
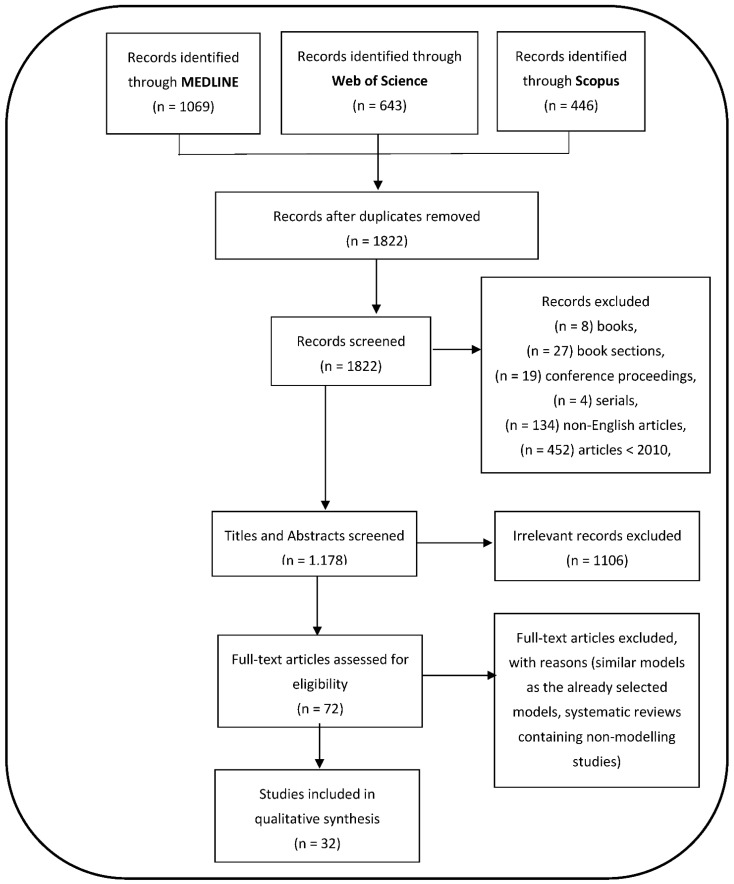
PRISMA flowchart for article selection process.

**Figure 3:**
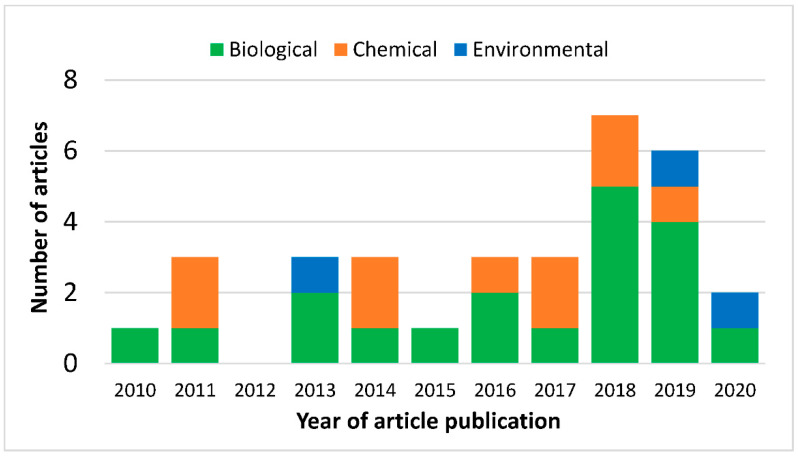
The yearly distribution of the number of selected published modelling articles with respect to the vector control types.

A total of 2158 articles were identified from the standard databases: MEDLINE—1069, WOS—643, and SCOPUS—446. Of these, 336 duplicated studies were found and subsequently removed. Of the remaining 1822 studies screened, 644 records were excluded. This exclusion includes 8 books, 27 book sections, 19 conference proceedings, 4 serials, 134 non-English articles, and 452 articles whose years were after 2010. The 1178 remaining articles were screened by reading the titles and abstracts. Of these, 1106 studies were excluded because they were deemed irrelevant, i.e., not containing information governing the inclusion criteria and the scope of the review. In total, 72 full-text study assessments were performed. Of these, we excluded 40 articles that either did not describe deterministic, stochastic, network models, or the vector control methods as a technique for dengue control and whose aim is closely related to the selected articles. Finally, 32 articles were selected and considered in this review (Figure 2). The distribution of these review articles by calendar year is shown in Figure 3. In Figure 3, more than half of the 32 selected modelling articles were published in or after 2017, i.e., over the past four years.

### 3.2. Distribution of Vector Control Modelling Articles

In this review, 32 modelling articles were considered and included in the study. These articles were published between 2010 and 2020 (Figure 3). The stacked bar chart in Figure 3 shows the distribution of the three different vector control strategies over the years. Beginning with the biological vector control, there is an increasing trend in the number of published articles, and this is mostly seen in the last two years. This increase may have been fuelled by the largest dengue viral outbreak in the Americas, southeast Asia, Europe, sub-Saharan Africa, and Oceania regions and by greater recognition of this novel strategy [71]. The number of publications addressing chemical methods of vector control remains approximately constant over the years, reflecting its ongoing dominant role in contemporary vector control [72]. Only three papers address environmental controls (Figure 3), and two of the three are from the last two years of publications in this study, suggesting this may be a growing area of interest [4]. Most (28 out of 32) of the overall articles selected are deterministic modelling studies of dengue transmission [32,34,35,37,38,39,40,41,42,43,44,46,48,49,50,51,52,53,54,56,57,58,59,61,62,63,64,65]. Three of the thirty-two articles are stochastic modelling studies [33,47,60]. Only one is a network modelling study [55].

Ten (10) of the 32 articles investigated the chemical method for vector control [33,37,46,47,48,49,54,56,61,62,63]; nineteen (19) articles described the biological method of control [32,34,35,38,40,41,42,43,44,50,51,52,53,55,57,58,64,65]; while only three (3) of the articles partially discussed the environmental method for vector control [39,59,60]. More than half (60%) of the selected modelling studies were categorised under biological control methods. The chemical methods of control articles were 31%, while the least (9%) was that of the environmental methods. We now discuss the vector control methods elaborately below.

## 4. Vector Control Methods

The selected dengue modelling studies have been characterised by the aims and objectives, year of publication, modelling methods, study location, vector control types, and summary of the articles to gain clarity (Table 1).

### 4.1. Chemical Control Methods

This method describes the use of a chemical solution, mixture, aerosol, or material to directly repel, expel, or, in some cases, kill arthropod vectors, such as mosquitoes [73,74,75]. Chemical control includes the use of pyrethroids for outdoor fogging [76,77], insecticides for indoor and outdoor residual spraying [17,78], insecticide-treated bed nets (ITN) [79,80], insecticide-treated house screens (ITHS) [81], and insecticide-treated door curtains (ITC) [82], and chemical larvicides, such as temephos, to directly kill or destroy mosquito vectors and their breeding sites [83,84]. 

Ten dengue transmission modelling studies in the selected articles incorporate chemical control methods [33,37,46,47,48,49,54,56,61,63]. Of these, eight were based on deterministic models (DM) [37,46,48,49,54,56,61,63], while the other two used stochastic modelling (SM) [33,47]. Some studies based on the DM featured vector populations only [63] or both human and vector populations [37,46,48,49,54,56,61], while SM studies considered coevolution dynamics of both humans and mosquitoes. For some of the DMs modelling both human and vector populations, the basic reproductive number (R0) with respect to dengue was computed and further illustrated that the disease-free equilibrium (DFE) is stable if it is less than unity (i.e., R0 < 1) [46,49,54,61] or in the presence of backward bifurcation where DFE may coexist with an endemic equilibrium [37]. A study estimated the value of R0 during the 2014 Guangdong Province dengue outbreak in China to be around 1.74, and after implementing impulsive vector control strategies, it reduced to 0.17 [61], while the R0 for the 2017 dengue outbreak in Pakistan was estimated to be approximately 2.65 [37] and another study estimated the R0 to be 8.16 [49]. One modelling study, alongside experimental data from Brazil, predicted that the dissemination of pyriproxyfen would, under some optimistic, realistic, and worse-case epidemiological scenarios (based on parameter modification, such as daily vector death rate, vector-to-human ratio, among others), reduce the values of R0 with respect to *Aedes*-borne viruses, such as dengue, Zika, and chikungunya (which was estimated to be between 3–45, i.e., range of optimistic to worse-case scenarios) by 100–1000 fold [46]. 

Almost half (13 out of 32) of the selected studies performed sensitivity analysis [33,35,37,38,42,43,44,53,54,57,59,61,65]. Four of the studies [35,37,59,61] used the Latin hypercube sampling (LHS) and partial rank correlation coefficients (PRCC) and provided evidence that the most effective parameters in curtailing dengue viral spread include reducing the transmission probability per contact of either infectious mosquitoes with susceptible humans or susceptible mosquitoes with infectious humans; mosquito recruitment and death rates; and human recovery rates [37,61]. Other highly sensitive parameters include the epidemic size, date of arrival of the infectious human and mean annual temperature [59]. One study used probabilistic sensitivity and threshold analyses and showed that if the cost of adult mosquito control was greater than 16 times that of larval control, all the adult vector control strategies were dominated [54]. Another study investigated the sensitivity of the vector model with respect to the parameter values used for the SIT method for dengue vector control and showed that shorter mosquito lifespans significantly prevent the disease from occurring; however, the disease could possibly persist (few cases) if the mosquito lifespan is less than its extrinsic incubation period [38]. 

To account for the robustness of the transmission models with respect to the corresponding biological implications, two studies [44,65] performed sensitivity analyses on a large range of parameter values and found that fitness cost and maternal transmission parameters are the main factors that determine the establishment of *Wolbachia*-infected mosquitoes in a wild-type population [44,65]. The effect of these factors could induce a backward bifurcation when the fitness cost is high [65]. A study by Li et al. investigated the robustness of the release strategy of the model for different values of CI. The authors showed that there was no significant difference between the different values of CI chosen and, as such, suggested excellent robustness [53]. Similarly, some researchers explored the uncertainty in asymptomatic cases of dengue viral transmission in Brazil. They simulated two scenarios with 50% and 100% transmission rates for asymptomatic individuals and found that there was a similar reduction in dengue cases for both rates [33]. A study that described the dengue transmission dynamics using boosted SIT approaches performed a sensitivity analysis on the model parameters and showed that for low release rates boosting the SIT methods reduced the elimination threshold the most [57]. Some of the modelling studies [46,47,48,61,63] revealed that the use of insecticides or fumigation significantly reduced the emergence and production of vectors (in particular, *Aedes aegypti* mosquitoes) and, in turn, reduced the burden of dengue [33,46,49,56]. However, continual long-term mosquito larvae control may prove ineffective due to the evolution of mosquito resistance [54]. 

Further, some modelling studies considered cost-effectiveness and benefits as part of their analysis [37,38,41,43,48,54,62,63]. Cost-effectiveness analysis—which includes the calculation of the infection averted ratio (IAR), average cost-effectiveness ratio (ACER), and incremental cost-effectiveness ratio (ICER)—describes the advantages and costs with respect to the control strategies, allowing one to identify the most effective strategy [37,38,48,54]. The authors in [48] investigated the cost–benefits of dengue control strategies via insecticide-treated bed-nets (ITN) and spraying interventions. They found that the combination of both strategies with low-cost weight was most effective in reducing dengue infections (IAR = 0.76, ACER = 5.65); however, the insecticide spraying strategy only was the most cost-effective (IAR = 0.71, ACER = 4.35) [48]. Another study investigated the potential costs per dengue case averted by the SIT control strategy based on the average cost estimates derived from SIT data. They showed that the yearly mean per capita cost of the vector control measures was USD 0.765. That is, the yearly control cost of the simulated two million people would be USD 1.53 million, and for about 5000 people, that would be USD 3825. This would save a large percentage of infection [38]. A study described 43 insecticide-based interventions together with the cost-effectiveness of different (larval and adult mosquito) control strategies and stratified the control efficacy into high (90% mosquito mortality [MM]), intermediate (60% MM), and low (30% MM) with annual application frequencies [54]. They showed that the high-efficacy adult vector control strategies dominated the larval control strategies because of the increasing resistance of larvae within the 5-year time horizon. Of the frequencies examined, twice annual control was the most efficient (ICER = USD 615), whereas six times annual control was the most effective (ICER = USD 1267), with the increased cost per disability-adjusted life years (DALY) saved meeting the willingness to pay threshold. This suggests that of all the vector controls, the one that most significantly reduced the DALYs lost was the high-efficacy adult mosquito control with six application frequencies [54]. The authors in [43] investigated the impact of the *Wolbachia*-based strategy in reducing the dengue burden and estimated over 330 thousand DALYs lost attributable to dengue in Indonesia. Of the DALYs lost, approximately a quarter is caused by a fatality, while the rest is due to disability [43].

A number of studies explored the optimal control strategies associated with the cost benefits of dengue control strategies [37,41,62,63]. A study of dengue where model outputs were validated with experimental data in Pakistan introduced two time-dependent control variables: the use of insecticide and vaccination [37]. They investigated the effect of costs on the cost weights of insecticide and vaccination use and found that as the cost of insecticides increases, the use of vaccination also increases. However, when vaccination decreased as a result of an increase in cost, an exiguous increase in the use of insecticide was observed. Although the two control strategies avert almost equal numbers of infections in the Pakistani region, this occurred as a result of the existence of a reciprocal relationship between insecticide cost and vaccination use [37]. Another modelling study that also described the minimum effort in mitigating dengue via suppressing the vector (female mosquito) population considered the production cost of SIT, insecticide application cost, and social (dengue disease-related) cost. They showed that the social cost is very important in reducing the female mosquito population and should be considered in controlling vectors responsible for transmitting dengue [62]. Further, researchers in [41] considered optimal control approaches for establishing *Wolbachia*-infected mosquitoes and compared two options: (a) the importance of both time and production cost of *Wolbachia*-infected mosquitoes; and (b) time is more important than the cost of production. Option (b) results in overpopulation of *Wolbachia*-infected mosquitoes as this is a negative side effect, while option (a) can annul the overpopulation effect as releases are suspended approximately 5.5 weeks earlier [41]. One study investigated mosquito reduction management and cost using temephos and fumigation. They showed that the most effective strategies in mitigating dengue burden with the cheapest cost when control was initiated with a small number of vectors together with a simultaneous combination of both temephos and fumigation control measures [63].

Some studies investigated optimal control strategies to avert disease burden [37,48,49,56,63] and the effect of seasonality [48,49,56]. A study investigated the optimal control strategies for insecticide spraying and the use of insecticide-treated bed nets (ITN) to mitigate human infections and intervention costs [48]. They showed that the most effective strategy in averting dengue infections is the combination of ITN and insecticide spraying. However, insecticide spraying alone (without additional ITN) should be implemented in areas of at least low seasonality, being both effective and the most cost beneficial. In the absence of seasonality (amplitude of seasonal force) for mild climate scenarios, ITN could not eliminate disease (within a year timeframe). However, combining ITN with insecticide spraying reduced the infection prevalence and led to no infection within five months. Wijaya and Gotz also investigated the application of optimal control models via two categories: chemical dissemination of aquatic mosquitoes (eggs and larvae) and fumigation of adult mosquitoes [63]. Results from numerical simulations suggest that, although maintaining fumigation instead of the use of chemicals, such as temephos, may be beneficial, combining the two control strategies most significantly reduced the vector (mosquito) population [63].

Another study, which sought to reveal two optimal timings at which pesticide is sprayed within the seasonal period in a year, investigated two optimization scenarios of pesticide spraying [49]. The first scenario described fixing the peak number of dengue-infected hosts at specific intervals and, afterwards, detecting the optimal timing through repetition of pesticide application timings that generates the minimum amounts of pesticide per application required. The second scenario inversely considered fixing the number of pesticides and minimising the dengue infection peak. It was shown via the first scenario that the optimal timing of the first pesticide application varies between 33–43 days, while the second remains approximately constant at 220 days before the peak of infection. However, the second scenario revealed that the optimal timing for the first pesticide application remains constant (around 28 days) while the second takes values between 232–281 days [49]. Two similar studies also investigated the optimal timing of insecticide fogging together with seasonality (where both wet and dry seasons were considered) [56,85]. The authors in [56] simulated four scenarios of the model, which include adding seasonality, endemic state, and different transmission intensities by systematically increasing the number of mosquitoes per person (that is, from low (2) to very high endemicity (15)). They concluded that the optimal timing of application oscillated between the beginning of the wet season (dengue season) and the prevalence peak [56]. This occurred because the timing could interfere with the exponentially growing epidemic. Further, researchers in [85] investigated the optimal timing of fogging, which includes the deployment of ultra-low volume (ULV) and targeted indoor residual spraying (TIRS), using different spraying strategies, such as yearly, biannually, or when the number of dengue cases exceeded the adaptive threshold of the average incidence. They used a simulation-based model to parameterize data from 2000 to 2010 in Iquitos, Peru. They showed, in general, TIRS has higher efficacy and averted more dengue infections than ULV. Of the different spraying strategies applied to both ULV and TIRS, the adaptive threshold strategy for TIRS is the most effective, with a 97% reduction in the number of infections from baseline and requiring fewer days of spraying (three quarters of a year) [85]. A sensitivity analysis was performed to explore how the adaptive threshold spraying strategies could be affected by delays in reporting or underreporting. They found that these delays do not affect the serotype-specific calibration of the model [85]. 

### 4.2. Biological Control Methods

Biological methods for vector control of dengue encompass the introduction of biological agents, such as small fishes, crustaceans, and bacteria [16,75,86]. These agents typically include larvivorous fishes [87,88], cyclopoid copepods [89,90], and *Bacillus thuringiensis israelensis* (BTI) [91,92,93]. Additionally, biological control methods may also include the alteration of the genetic materials of vectors, thereby inhibiting them from transmitting the dengue virus [16]. This subclass includes sterile insect techniques (SIT) [94,95], genetically modified mosquito (GMM) methods [96,97], such as the release of insects carrying a dominant lethal (RIDL) gene [96,98] and *Wolbachia* bacterium introduction (WI) [15,22,23,29,99].

Prior to formulating models to biologically control vectors fuelling the transmission of dengue, it is worth mentioning, in general, some factors to be considered in governing model interests and initiation. These factors include describing the biological agents (vectors) to be used [16,36,49,53,86,88], understanding ecological patterns between the vector and dengue virus [15,83,100], and identifying the methods of control [15,29,67,95,96]. Different model structures account for the biological vectors used by modelling vector-only transmission dynamics involving the dengue virus [29,40,64]. These models account for the competitive interaction between wild-type and biologically infected vectors, in particular, mosquitoes [38,42]. Of the three factors, the ecological patterns between the vector and the virus can be modelled using human–vector transmission models [52,57,101], which capture the interaction between the viral-infected mosquitoes and uninfected humans and vice versa. These control methods are considered by incorporating a control type, such as *Wolbachia*-based control, that may consider complex features, such as CI and IMT effects in vectors [35,95,98,99]. These are some of the complexities in model structures used for different forms of biological controls.

Dengue models formulated by investigating the effects of the biological methods of vector control are described in 19 studies [32,34,35,38,40,41,42,43,44,50,51,52,53,55,57,58,62,64,65]. Except for one [55], which is a network model (NM), all of the other 18 studies use deterministic models (DM). Of all the 19 modelling studies, seven articles [35,38,42,43,52,55,57] model the interaction between human and vector populations, while 12 studies [32,34,40,41,44,50,51,53,58,62,64,65] model the vector population dynamics, where the vector population in all cases is considered in the presence of a biological control mechanism, such as SIT, GMM, or *Wolbachia* introductions. Presently, the biological method of dengue vector control is the most commonly modelled and analysed as compared with about a decade ago (prior to the successful introduction of some biological techniques, such as WI), when mostly chemical control methods were considered [66]. 

Different studies have recognised key determinants of success-mating competitiveness of SIT versus wild-type mosquitoes in combination with other control methods and recruitment and release rates to control dengue viral infection [32,34,40,55,57,62]. These DM of SIT studies [32,34,40,62] model vector population dynamics and investigate release rates and sizes. Another DM study [57] considered both vector and vector–human population dynamics, and one NM study [55] only described human–vector population dynamics. These models involved state variables which may include young and adult sex-structured vector populations. One similar study assumed that the male mortality rate is higher than that of the female [32]. These studies considered constant [32,34,40] and periodic [32,34,62] releases of sterile insects to achieve elimination. For the constant releases (usually sterile males), the number of sterile mosquitoes released at the initial stage of the release timeframe is constant. This demonstrates that every solution of the system tends to an equilibrium point (especially disease-free equilibrium) for the constant release of sterile mosquitoes, even in the presence of a time delay in the developmental stage of the uninfected mosquitoes [34,40]. For periodic releases, control is achieved at a lesser cost compared to constant release control [34].

A study [40], which modelled the interactive competitiveness between wild-type and sterile mosquitoes, considered and analysed the delay in time to releasing sterile mosquitoes. They showed that the delay imposed while releasing the sterile mosquitoes at a constant rate does not significantly affect the system dynamics. However, if both wild-type and sterile mosquitoes are released at the same rate, there is a profound effect on the system dynamics, especially as the delay in release time increases as a result of Hopf bifurcations [40]. In contrast to constant release, delay in periodic releases of sterile males (i.e., release rate proportional to the wild mosquito population) may greatly affect the system dynamics. As the time delay increases, the system’s solution may show an oscillatory behaviour through Hopf bifurcations. At this point, both the sterile and wild mosquito populations can coexist [40]. 

Several SIT control approaches require sterile mosquitoes to compete almost equally with their wild counterparts to be effective, as their effectiveness may depend on the size of the wild populations [34]. The study [34] further suggested that the mating competitiveness of the SIT control method should tend to one (as good as the wild-type) to boost effectiveness. Otherwise, the SIT efficacy may diminish, provided there are existing wild-type mosquitoes. Another study [57] showed that when the SIT is supplemented with pupicide pyriproxifen (PP), it could increase the effectiveness of the intervention by averting over 95% of the total rollout and, in turn, decrease the dengue burden. Additionally, a network model [55] revealed that SIT application could be successful depending on the rate of recruitment and coupling strength of the migration parameter. 

The modelling of genetically modified mosquitoes (GMM), such as the release of insects carrying dominant lethal (RIDL) methods, are analysed in three studies [38,58,64]. A dynamic model that accounts for the RIDL release of pupal and adult mosquitoes was unveiled via simulations that, for regularly recurring releases, the most effective RIDL approach is evident when only adult-carrying RIDL mosquitoes are released every day [64]. The adult-only RIDL mosquito approach outperforms both pupal and combined mosquitoes’ releases because the adult male RIDL mosquitoes are already sexually matured and, as such, would perform well in increasingly maintaining the RIDL mosquitoes after release until the next day’s release. Unlike adult RIDL releases, pupal-only RIDL releases would require that the pupae gradually develop into adult males and therefore are affected by high mortality between the pupa to sexually active adult time, causing a disadvantage. Whereas, for the long-term suppression of wild population scenarios with infrequent RIDL releases, the combination of pupal and adult mosquitoes’ release could maintain and sustain suppression every week when compared with pupal- or adult-only releases. Similarly, about 1.9 million combined mosquitoes’ release (73% pupae and 27% adults) was able to maintain suppression, while pupae- or adult-only mosquitoes’ release could maintain suppression if the population sizes were increased to 2.7 and 2.8 million, respectively [64]. Further, another model considered a GMM “reduce and replace” (RR) technique that introduced insects possessing genetic features, which included female-killing and antipathogenic attributes [58]. The authors showed that the continuous release of RR mosquitoes resulted in a long-term decrease in the overall density of the vectors (mosquitoes) [58]. When a proportion of RR male-only mosquitoes were released for a year, the population density of the adult female mosquitoes decreased with a more rapid reduction in the density of the competent vectors than the population density of the total female mosquitoes. The competent vector density decreased rapidly due to an increase in the frequency of the antipathogenic allele. However, when the release ceased, the female population recovered to its initial size, but the competent vector remained at an insignificantly low density [58]. Considering the release of RR female mosquitoes and comparing with male-only and both-sex mosquito releases for 100 days with a release ratio of one, the female-only releases mostly reduced the total adult female wild population. Although the total female population surged initially as a result of releasing more RR females, the total density of adult females was effectively reduced for longer periods of time. Concisely, it was shown that suppressing the vector population density would be dependent on release proportion and duration and adult female mosquitoes’ inclusion in the GMM releases [58]. Increasing the fitness cost associated with the antipathogenic gene for a year simulation of male-only RR rollout at a release ratio of two led to a reduction in the competing vector density [58]. Modelling the effect of releasing RIDL from the cost-effectiveness perspective [38] showed that the RIDL control technique could quickly eradicate dengue at a low cost and was, therefore, highly cost-effective. 

From the study articles selected for this review and then stratified into biological vector control so far, *Wolbachia* control strategies are the most modelled and have been analysed to inform the effective control of *Aedes* mosquito vectors to mitigate dengue burden [35,41,42,43,44,50,51,52,53,65]. *Wolbachia*-based control is the introduction of the intracellular bacterium *Wolbachia* into arthropods to suppress vector populations, disrupt arboviral transmission, or both [29]. *Wolbachia* infection is transmitted maternally (that is, from the adult female arthropod to the offspring). There are various strains of *Wolbachia*, such as *w*Au, *w*Mel, and *w*Pip, amongst others. *Wolbachia* possess some features that may depend on the strains, such as uni- or bi-directional cytoplasmic incompatibility (CI), the phenomenon that causes incompatibility between the sperms and eggs of arthropods (mosquitoes) resulting in unviable offspring; imperfect maternal transmission (IMT); viral blockage; *Wolbachia* infection loss; and mosquito fitness cost [29]. Sex-structured models accounting for the interactive competitiveness of wild-type and *Wolbachia*-infected mosquitoes were described in [41,44,53,65]. Some models have investigated *Wolbachia*-carrying mosquito features, such as fitness effects, IMT, viral blockage, CI, and *Wolbachia* loss [30,41,42,45,51,65]. These features have been suggested to affect the spread, establishment, and dominance of *Wolbachia* infections in mosquitoes [30,45,101]. One study showed that the evolution of a complete CI could drive the successful invasion of *Wolbachia* in a wild-type mosquito population; however, incomplete CI by genetic evolution may compromise successful invasion [51]. In other words, the authors revealed that the successful establishment or failure of *Wolbachia*-infected mosquitoes might rely on the selected *Wolbachia* strain [51]. 

Other human–vector dynamical models in the presence of *Wolbachia* have been analysed as these models, together with experimental data, have provided insights into how the presence of *Wolbachia*-infected mosquito rollouts have significantly reduced dengue disease [35,42,43,50,52]. One of these studies [43], focusing on dengue infection and *Wolbachia*-infected mosquito rollout dynamics in Indonesia, used the combination of multiple modelling methods together with available data from Indonesia to show that approximately 7.8 million dengue cases were estimated to be symptomatic in 2015. However, this analysis may be an overestimation as it is highly sensitive to the assumed under-reporting rate, where about five million cases were estimated to have individually managed the symptoms via informal healthcare services. Additionally, of the total estimated symptomatic cases, only 14.1% were estimated to have been hospitalized, resulting in over three thousand deaths [43]. The researchers in [43] also estimated that the *Wolbachia* rollout program conducted in Indonesia averted 86.2% of dengue cases over a year. Similarly, another modelling article used an estimated transmission rate of 0.1648 new human transmissions per dengue-infected mosquito per day by fitting a deterministic model to experimental data from a northern Queensland city, Cairns, in Australia. The authors estimated an 80% decrease in overall dengue cases after a *Wolbachia* rollout [42]. Further, they showed that for weekly introductions of *Wolbachia* in Cairns, half of the dengue cases were reduced for a year, while about 60% were reduced for quarterly time periods. The researchers in [42] further showed that the duration of dengue outbreaks could be decreased by between 2 and 6 weeks yearly in the presence of *Wolbachia*-infected mosquito rollouts based on the seasonality strength. This decrease may have been caused by a reduction in the mosquito’s lifespan [42]. The study [52] also described modelling the use of *Wolbachia* for dengue control. They showed that infecting *Aedes* mosquitoes with *Wolbachia* bacteria decreased the basic reproductive number for dengue virus and, in particular, for the *w*AlbB *Wolbachia* strain, the reproductive number is reduced by around two-thirds, which would be sufficient to prevent epidemic outbreaks [52].

### 4.3. Environmental Control Methods

Environmental control programs focus on the reduction of mosquito breeding and reproduction via the media of modification to the surrounding environment. These media include installing efficient piped water supplies and good drainage systems; emptying, covering, or destroying stagnant waterlogged cans and containers; practising proper environmental hygiene (cleaning of the environment such as mosquito breeding sites); and implementing waste management schemes. Additionally, environmental factors, such as seasonal variation and changes in temperature, may also serve as environmental modifications of vectors to mitigate their abundance. Of all the vector control methods, environmental control does not pose environmental contamination risks as it predominantly entails common hygienic practices and maintenance, addressing seasonal fluctuations in cases. Its impact can be lifelong and does not require further investments for sustainability. 

Mathematical models of environmental control studies have sparsely been formulated as these models are not often described (in just three of the selected articles) [39,59,60]. The deterministic models in [39,59] showed that seasonal variations could affect the dengue epidemic dynamics; that is, autumn and summer seasons could greatly increase dengue transmission in the presence of an infectious individual within a short time period [59]. This suggests that large outbreaks of dengue could have been fuelled by warm temperatures [59]. A model [39] that considered seasonal variations via periodic forcing in the vector density and the impact of vector control methods in the 2005 Singapore dengue outbreak estimated the basic reproductive number to be 1.363 [39]. The authors showed that dengue infection would not persist except if the recruitment rate is more than the ratio of the periodic vector recruitment rate with a yearly period to the square of the basic reproductive number [39]. This showed that the basic reproduction number, under periodic environment (or asymptotic behaviour of the system—bifurcation), described the threshold for disease persistence. They also revealed that the dengue incidence, which occurred in the 10th month of each year (2003–2005), was described by a lag of 4.2 months with the highest mosquito density [39]. Similarly, another model, together with experimental data from Funchal, Madeira Island, accounts for seasonality via varying temperature over time by periodic forcing in the dengue transmission in Madeira Island, Portugal [59]. Considering the different simulated arrival dates of an infectious individual into the population of the uninfected/susceptible, it was revealed that an epidemic outbreak is expected to occur between July and November each year [59]. Therefore, with an infected individual arrival time in August and October, the shortest and longest epidemic time simulated was 93 days and 411 days, respectively. For the shortest epidemic time, approximately one-tenth of the susceptible population was infected, while 3.4% of susceptibles were infected in the longest epidemic time [59]. A general multi-patch model of dengue dynamics together with experimental data from Kolkata, India, revealed that control methods with higher environmental persistence, such as the treatment of surface and materials (TSM), mostly decreased dengue cases as compared with the use of ultra-low volume (ULV) spray of insecticides and lethal ovitraps (LO) [60]. Specifically, the comparison between the single applications of the three strategies: the use of ULV, LO, and TSM showed reductions of 2.9%, 48%, and 49.1%, respectively, with TSM ranking highest. Considering pairwise and three-way comparisons of the control methods, any combination with TSM ranked highest as the three-way combination reduced 72.7% of the total cumulative cases [60]. In all, the above review suggests that environmental vector control modelling studies have lots of potential but are currently under-investigated, and, therefore, more modelling studies need to be conducted to account for the environmental vector control impacts towards the eradication of dengue.

### 4.4. Quality Assessment of Study Results

The selected studies possessed an Assessment of Modelling Studies (AMS) score range from 66.67–91.67% (Table 3). Thus, all scored at least a ‘High’ methodological quality (cut-off for high is 65%). Additionally, of the 32 studies included, 13 scored above 80%, as these studies fell into the ‘Very High’ category on the Assessment of Study Quality (ASQ). In all, the selected studies provided in detail the description of model structure, methods and validation, parameter specifications, assumptions made, intervention comparators and quality of data, and uncertainty/sensitivity analysis. 

**Figure 4:**
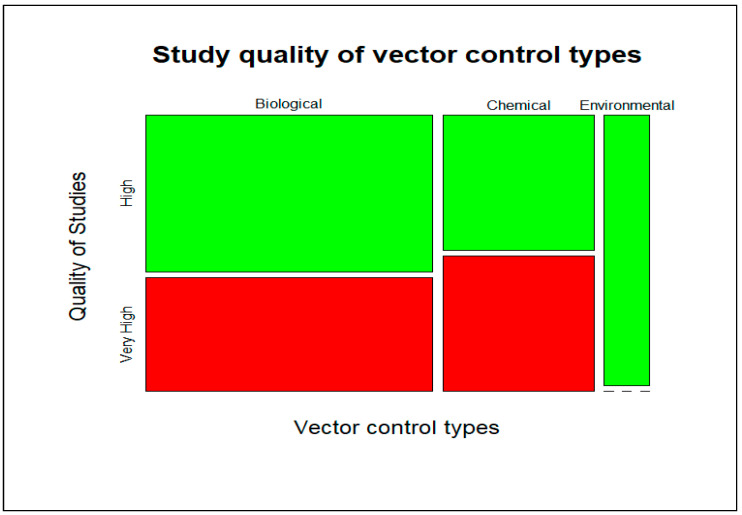
Mozaic plot showing the distribution of the bounded area of study quality of the vector control types.

To further investigate if there was a difference in the quality of the studies (Very High and High) across the different vector control methods (chemical, biological, and environmental), a bivariate analysis was performed using Fisher’s exact test. Our analysis showed that there is no significant difference in the study quality (Very High and High) of the different vector control methods (*p* = 0.4). In other words, there is no statistically significant difference in the proportion of ‘High’ versus ‘Very High’ quality studies across the vector controls (Figure 4). Given the relatively small number of studies investigating environmental control (3 out of 32), the ‘High’ rating assigned to this category should be interpreted with caution.

## 5. Discussion

The mathematical modelling of dengue transmission can be useful in understanding disease dynamics [102,103,104]. It is a significant tool that can assist in predicting and curtailing outbreaks of dengue disease and help in reducing transmission of infection and mitigating the dengue burden [35,42,47,54,59,60]. Overall, our study reviews the effectiveness of mathematical modelling of different vector control approaches to reduce the spread of dengue disease. In this review, we identified 32 articles that met our search criteria. We then stratified the selected articles into the modelling types: deterministic, stochastic and network modelling methods. The articles mainly consisted of deterministic modelling methods for dengue vector control (88% of the total selected studies). Other modelling approaches, such as stochastic and network modelling, shared 9% and 3% of the total selected studies, respectively. Based on the vector control approaches, we grouped the selected articles into three vector control approaches: chemical (11), biological (18), and environmental (3).

Modelling studies demonstrate that the chemical vector control methods, such as the use of insecticides for outdoor fogging or indoor residual spraying, insecticide-treated bed nets (ITN), insecticide-treated house screens (ITHS), and insecticide-treated door curtains (ITC) for dengue transmission, could be highly effective in reducing the burden of dengue when scaled up [33,46,49,56]. They also showed that when the dengue burden is high, chemical controls are best used in combination, while a single control technique, such as insecticide spraying, may be adequate for areas with low endemicity [48]. One important prediction from modelling is that long-time usage of this method could fuel mosquitoes’ resistance to the chemicides and then result in a less effective and efficient control strategy [54]. These modelling results need to be considered in addition to the known threat to the environment via contaminating water bodies and air pollution [16].

On the other hand, biological vector control methods are gaining global popularity and usage as some of these methods are self-sustaining [29,30]. Accordingly, various mathematical models accounting for the biological control of vectors to mitigate dengue spread have been formulated in the last decades [32,34,40,55,57,62]. These modelling studies consider the transmission dynamics of releasing sterile insect techniques (SIT), genetically modified mosquitoes (GMM) and *Wolbachia*-infected mosquitoes (among other interventions) to curb the spread of dengue infection. Our review has presented an understanding of SIT techniques and how they could be very effective in controlling dengue infection; however, when boosted with pupicide pyriproxifen (PP), they could greatly reduce the number of sterile males required to eliminate the mosquito population [57]. This could only be established in areas with high but not low mosquito densities. According to a network model, SIT could be very effective in reducing dengue viral infection as that would depend on the rate of recruiting sterile mosquitoes, migration parameters and coupling effect [55]. For GMM methods, such as RIDL techniques, which could be less expensive to conduct [38], the effectiveness of these methods could depend on the proportion of RIDL release, duration of release, and, most especially, the inclusion of adult-RIDL female mosquitoes [58]. 

*Wolbachia*-infected mosquito rollouts have been very effective in averting dengue cases, with an estimated reduction of over 80% in countries such as Indonesia and Australia [42,43]. Since then, there have been increasing numbers of models addressing transmission dynamics and features that drive successful strategies, with 10 of the 18 models on biological methods focused on *Wolbachia*-based mosquito control. The effectiveness of *Wolbachia*-carrying mosquitoes is dependent on reproductive advantage CI, fitness effect, maternal transmission, and viral blocking strength [22]. Furthermore, *Wolbachia*-infected mosquito release programs could also decrease the duration of feasible outbreaks of dengue infection by a month and a half [43].

Regarding the mathematical modelling of environmental control methods, there are few articles describing the modelling impact. Although some of these models are conjoined with other control methods, such as chemical and biological methods, model outcomes suggest that environmental interventions—for instance, treating surfaces and materials and seasonal variations could have a greater impact in reducing dengue cases when compared to chemical methods, such as insecticide spraying without the attendant environmental contamination [39,59,60]. This sparse modelling work is encouraging, and future studies on environmental actions alone and in combination with other control measures are needed.

### Strengths and Limitations

This review relied on the modelling results of articles taken from the extensive database search describing the vector controls of dengue transmission dynamics models. These models showcased the different control techniques in mitigating dengue viral transmission. As far as the authors are aware, this review is the first to explore the present understanding of vector control approaches and the effective role of mathematical models in mitigating or eliminating dengue. However, the selected articles were not evenly distributed as more than half of the studies were from 2017 onwards (Figure 3). This may have been a consequence of the severe dengue outbreaks in the Americas, and some parts of South East Asian region from 2018 to 2019, arousing interest in vector control and modelling studies [71]. Further, only published English journal articles were considered as non-English studies were excluded to avoid oversight. Other referencing types, such as book sections, conference proceedings, and serials, were excluded as they did not contain sufficient detail to assess the studies. In addition, the other referencing types are approximately 3% of the searched articles after removing the duplicates, and, as such, their exclusion has an insignificant impact. We have only considered dengue transmission models via three major control types (chemical, biological, and environmental) in this work. Of these control types, environmental control studies constitute relatively few (9% of the selected studies) and that would have had an impact on the results, possibly creating some biases in the evaluation. Although there is an emerging control method, mechanical control, mathematical models are still sparsely formulated and analysed in this regard. Essentially, the AMS tool used in appraising the included articles relies in part on the authors’ knowledge and, as such, could create grounds for possible bias [69]. Overall, there is a chance for information bias as some articles may not have been included in the databases used for this research study. 

## 6. Conclusions

In summary, our study, based on the selected published articles, provided a detailed understanding of all three methods of vector controls and their effectiveness. However, the magnitude of their effectiveness has some dependencies. The chemical method has some drawbacks based on the evolution of vector resistance resulting in decreased efficacy of these methods. The biological method could be a self-sustaining form of control as trans-infected mosquitoes (with *Wolbachia*) could pass on the *Wolbachia* infections to the offspring, thereby, inhibiting the transmission of dengue. This has been shown to be very effective; however, factors such as seasonality and heatwaves could reduce the effectiveness through loss of *Wolbachia* infection in mosquitoes. Environmental control methods have lots of potential but are currently under-investigated; therefore, there is a need to further model environmental parameters, such as healthy drainage systems, covering of water containers, and good hygiene, to inform the impact on the dengue burden. In all, there is a strong need to consider the combination of the three methods of vector control via mathematical modelling studies to evaluate the impact on the eradication or elimination of dengue disease at large.

## Figures and Tables

**Table 1:** Characteristics of selected articles describing the year, aims and objectives, modelling methods, settings location, main vector control method and summary of studies. DM → deterministic model, SM → stochastic model, and NM → network model. C → chemical control, B → biological control, and E → environmental control. n/a→not available.

S/No.	Reference	Year	Aims and Objectives of Study	Modelling Methods	Settings	Vector Control Technique	Summary of Findings/Conclusion
1	Abad-Franch et al. [46]	2017	Explored the mosquito-disseminated larvicide pyriproxyfen for vector control via arboviral blockage	DM	Brazil: Manacapuru	C	Following the mosquito-disseminated insecticides (pyriproxyfen), there were drastic decreases in the emergence and catch of adult and young *Aedes* mosquitoes, respectively. This reduction inhibited the transmission of *Aedes*-borne viruses, such as dengue, chikungunya, and Zika.
2	Agusto and Khan [37]	2018	Developed a deterministic dengue virus transmission model and parameterized it using 2017 dengue outbreak data in Pakistan. A sensitivity analysis was conducted, and optimal control theory was applied.	DM	Pakistan	C	There is a strong reciprocal relationship between vaccination and the use of insecticides. Nonetheless, the use of insecticides slightly increases when there is a decrease in vaccination levels as a result of an increase in cost. Application of the two time-dependent controls derived from the sensitivity analysis could decrease the total number of infected mosquitoes and humans.
3	Alphey et al. [38]	2011	Combined epidemiological models and mosquito population dynamics to investigate the effect of releasing RIDL (release of insects carrying a dominant lethal) on dengue virus transmission.	DM	n/a	B	Having derived a preliminary estimate of the potential cost-effectiveness of vector control, it was predicted that the genetic control technique could swiftly eliminate dengue disease from a human community at a very low expense.
4	Andraud et al. [39]	2013	Developed a simple periodic-forced vector-host model. This model was based on a previously formulated model that investigated the impact of vector control techniques during a dengue outbreak in Singapore in 2005. The model in this work considered the seasonal variations in vector density and estimated the parameters using dengue fever incidence data from August 2003 to the end of 2007.	DM	Singapore	E	After fitting the model outputs with the dengue incidence data, there was a good fit, which suggests that the impact of seasonality on dengue transmission dynamics was highly essential, even though the model did not consider the complex life cycle of the vector. Additionally, the seasonal fluctuations of the mosquito vector population occurred in phase with the variations in temperature. This signified a strong climatic effect on the vector abundance, thereby affecting the dengue virus transmission dynamics.
5	Barmak et al. [47]	2014	Presented a stochastic dynamical model for the transmission dynamics of dengue. This model accounted for the coevolution of human hosts and the spatial *Aedes aegypti* dynamics.	SM	n/a	C	For insecticide spraying techniques with different efficiencies, it was observed that the most efficient fumigation strategies could be effective during a dengue virus outbreak. Also, isolating infected humans with high compliance levels is an effective strategy; however, imposing restrictions on their movement is not likely to be effective. Therefore, combining fumigation and infected human isolation during a dengue outbreak would be a suitable strategy for mitigating the outbreaks.
6	Bliman et al. [32]	2019	Proposed a sex-structured model that captured the constant and periodic impulsive releases of sterile male *Aedes* mosquitoes in the hopes of eliminating wild-type mosquitos. This model serves as a foundation for vector control strategies.	DM	n/a	B	A mixed control strategy that requires the combination of open- and close-loop outputs that produce the best results regarding the total number of releases of sterile male mosquitoes to be effectively rolled out during the rollout program and the time required to achieve elimination.
7	Buonomo and Della Marca [48]	2018	Considered a mathematical model accounting for the use of insecticide-treated bed nets (ITN) by humans. The effect of seasonality, together with some varied rainfall and mean temperature scenarios, was investigated. The optimal control problem was used to mitigate the number of infected individuals, and a cost-effectiveness analysis was conducted to assess the most appropriate strategy for the elimination of dengue infection.	DM	n/a	C	The cost-effectiveness analysis showed that the benefits of the cost of intervention efforts were influenced by the shift in the periodic amplitude of the seasonal fluctuation. In general, of all the combination strategies for dengue disease control via its vectors considered, the most effective, averting the highest proportion of infections, is the use of ITN and insecticide spraying techniques. However, for areas with a low seasonality effect, only insecticide spraying campaigns should be conducted in the dengue control program as this is beneficial in terms of cost.
8	Cai et al. [40]	2018	Considered an interactive dynamical model of wild-type and sterile mosquitoes and accounted for the delay of the growth stage of the wild-type mosquito population. An analysis of the effect of the time delay of releasing sterile mosquitoes in two different rollouts was performed.	DM	n/a	B	At a constant release rate of sterile mosquitoes, the delay poses an insignificant effect on the system dynamics, and all the solutions of the system tend to an equilibrium point. At a release rate of sterile mosquitoes proportional to that of wild-type mosquitoes, the delay exhibits a significant effect on the system dynamics via some parameter ranges. For a small delay, the solutions tend to an equilibrium point. However, as the delay increases, the solutions of the system possess oscillatory behaviour by way of Hopf bifurcations.
9	Campo-Duarte et al. [41]	2018	A sex-structured population model was proposed describing the interaction between uninfected (male and female) and infected mosquitoes (via deliberate transinfection) with the *w*MelPop-*Wolbachia* strain in the same region. This model incorporated the natural introduction of the control or decision variable and introduced the optimal control approach to capture the dynamics of the *w*MelPop *Wolbachia* infection of the uninfected *Aedes aegypti* mosquito population. This was a targeted quest at estimating the number of *Wolbachia*-infected mosquitoes to be released in daily control action.	DM	n/a	B	The release policies derived from the model results, which are also consistent with Yeap et al. (2014), recommendations: (a) The release of *Wolbachia*-infected mosquitoes should be of considerable quantities; (b) releases of *Wolbachia-infected* mosquitoes should occur for a long time period; (c) the *w*MelPop *Wolbachia* strain invasion is only likely feasible in relatively isolated mosquito populations. Additionally, the method derived in this study can be advantageous to vector control interventions such that if the population density of wild-type mosquitoes is minimized at earlier stages by other control measures, such as SIT and insecticide spraying, the invasion of the *w*MelPop *Wolbachia* strain and replacement of wild mosquitoes can be swiftly attained at a low cost.
10	Chavez et al. [49]	2017	Presents a SIR model accounting for vector–host transmission dynamics and vice versa. The model incorporates pesticide control and seasonal variations of vector resurgence and disease transmission rates. Also, the effectiveness of the control strategy is investigated.	DM	n/a	C	Upon investigating the seasonal fluctuations, it was revealed that the timing of the applications of pesticides was highly influential in controlling dengue viral infection, i.e., in the required amount of pesticide to achieve tolerably moderate levels of infection. Also, time variations in the second pesticide application showed induced destabilization caused by a periodic-doubling bifurcation. Therefore, the solution within a year period loses stability, and a class of stable solutions within a two-year period occurs. Hence, the numerical investigations showed that avoiding the two-year periodic solution is best due to the drastic increase of dengue viral infections during the period.
11	Hancock et al. [50]	2016	Proposed a mathematical model to explain the transmission dynamics between *Aedes aegypti* mosquitoes and the intracellular bacterium, *Wolbachia*, which accounts for larval density-dependent fluctuation in fitness components of *Wolbachia*-infected and wild mosquitoes. This model was applied to study *Wolbachia* field releases and revealed how *Wolbachia* invasion end results could be highly dependent on the severity of the population density-dependent competition at the rollout locality. Following *Wolbachia* rollout programs, the period for establishing *Wolbachia* in the wild mosquito population can differ by over two years as this depends on the relative mosquito fitness of the field and laboratory conditions.	DM	n/a	B	The investigated models incorporating larval density-dependent demographical variation in mosquito traits are effective in elaborating *Aedes aegypti* mosquitos and *Wolbachia* dynamics in experimental mosquito populations. These models highlight the strong effects of mosquito density-dependence on *Wolbachia* dynamics in the field as well as help in controlling arboviral transmissions, such as Zika, dengue, and chikungunya, via the effective use of *Wolbachia.*
12	He et al. [51]	2017	Proposed multi-scale modelling incorporating the combination of a birth-pulse model with a genetically induced discrete model for the allelic frequencies. This model described the invasive spread of *Wolbachia* infection in mosquitoes resistant to CI.	DM	n/a	B	The results showed that the strategy for population eradication might not be actualised. However, a population replacement strategy may be feasibly realized with success to sensitive or resistant alleles. The failure or success of population replacement by *Wolbachia* may be dependent on the appropriate *Wolbachia* strain selected. Also, *Wolbachia*-induced parameters may cause catastrophic shifts in the stable states of the model system and may affect the rate of population replacement and density of wild mosquitoes.
13	Hughes and Britton [52]	2013	Developed a mathematical model used to describe the Human-mosquito dynamics in the presence of *Wolbachia* infection. The model further accounts for the introduction of *Wolbachia*-infected mosquitoes, which serves as a potential control measure for dengue transmission.	DM	n/a	B	The model results showed that the *Wolbachia* bacterium has the potential to control dengue transmissions in regions of moderate endemicity (that is, when the reproductive number, R0, is not too large). But if R0 is very high, *Wolbachia* can only have a slight effect on the population as it can only reduce but not eradicate the transmission of dengue. Moreover, if control strategies, such as mosquito population reduction, are adapted, combining the introduction of various strains of *Wolbachia* that completely inhibit dengue transmission may be worthwhile.
14	Li and Liu [53]	2018	Established a sex-structured model with birth pulse and investigated *Wolbachia* invasion dynamics and spread into the *Aedes* mosquito population. Additionally, it also studies the release strategies of *Wolbachia*-infected mosquitoes in wild mosquito populations.	DM	n/a	B	The modelling results showed that perfect maternal transmission drives a successful invasion of *Wolbachia* infection in mosquitoes. However, in the case of imperfect maternal transmission, either a partial replacement of the *Wolbachia* infection or *Wolbachia* extinction may occur. Further simulations revealed that the partial success of the *Wolbachia* replacement strategy is dependent on the number of initial *Wolbachia*-infected mosquitoes present.
15	Luz et al. [54]	2011	Developed a model describing the transmission dynamics of dengue that accounts for the evolution of insecticide resistance and immune responses in humans. In line with this, the dengue health burden of disability-adjusted life years was measured, and a cost-effectiveness analysis of insecticide control use was performed. Also, sensitivity and threshold analyses were performed to investigate the uncertainties of the parameters used in the results.	DM	n/a	C	Continual yearlong larval control can be ineffective at fuelling an increase in the burden of dengue epidemics as a result of the evolution of insecticide resistance and herd immunity loss. Additionally, six annual high-efficacy adult vector control applications have a cost-effectiveness ratio that may align with that of the WHO’s laydown standard.
16	Marini et al. [33]	2019	Developed a stochastic transmission model, which accounted for the geographical distribution of *Aedes* mosquitoes and human population and spatial transmission dynamics of dengue in Porto Alegre, Brazil. This model described the estimation of dengue cases that were avoided by ultra-low volume (ULV) insecticide spraying in the study region.	SM	Brazil: Porto Alegre	C	It was shown that a quarter of all the symptomatic cases were averted by insecticide spraying and low-income-induced *Aedes aegypti* mosquito death decreased intervention performance, as almost half of the mosquito population was killed by insecticide spraying.
17	Mishra et al. [55]	2018	Proposed a network model that described the host–vector dynamics in *n* patches to control dengue transmission. In this case, the control was based on sterile insect techniques (SIT). The required R0s were computed, and the existence and stability criteria for the steady states were analysed. Bifurcation effects were also investigated in relation to the disease-free and endemic equilibrium for an isolated patch.	NM	n/a	B	Following the analytical and numerical solutions, it was shown that dengue could be controlled in a network by adopting SIT in only one patch as it is required less to apply SIT to the whole network. This could be done by patch coupling. The applicable success of SIT relies on the coupling strength of the migration parameter and the recruitment rate of the sterile mosquito population.
18	Ndii et al. [42]	2016	Developed a mathematical model to investigate the effect of an endosymbiotic intracellular bacteria, *Wolbachia*, on the transmission dynamics and seasonality of dengue disease. The study focused on areas where dengue is not endemic but can spread as a result of human movement, especially with dengue imported cases.	DM	Australia: Cairns	B	The results of the study showed that *Wolbachia* decreased the total dengue case number by about 80%. Also, dengue outbreak times could be reduced by approximately 1.5 months annually in the presence of *Wolbachia*. The most significant effect was obtained when the seasonal force amplitude was low. Furthermore, the benefits of *Wolbachia* were dependent on the transmission rate.
19	Oki et al. [56]	2011	Formulated an SEIR model for dengue transmission capturing seasonal changes in mosquito lifespan and the optimal timing of insecticide fogging to mitigate the dengue disease burden in several wet season scenarios. Also, the assessment of insecticide fogging was simulated and studied at low and high levels of dengue endemicity over a 500-year time period producing an endemic state.	DM	n/a	C	The results showed that seasonal variation and the level of transmission intensity largely influenced the optimal timing of insecticide fogging and its impact. Insecticide fogging applications at optimal timing could control a substantial number of dengue virus cases.
20	O’Reilly et al. [43]	2019	Used the combination of multiple modelling methods for estimating the dengue disease burden to predict the dengue national case burden stratified by disease severity. Three different sources of data were used to map the spatial distribution of disease burden. Following a national release program of *Wolbachia*, the estimation of decreased dengue cases was performed using a collection of transmission models.	DM	Indonesia: Yogyakarta city	B	The results showed that about 7.8 million were estimated to have symptomatic cases of dengue in Indonesia in 2015. This estimated number of cases was related to about 3.23 thousand DALYs. The majority of the burden was due to underreporting as some asymptomatic or less severe dengue patients sought medical attention or had difficulty with disease diagnosis, respectively. The implementation of the national *Wolbachia* rollout program was estimated to significantly decrease dengue cases by 86.2% over the long term.
21	Pleydell and Bouyer [57]	2019	Modelled the dynamics of *Aedes* mosquito populations incorporating the SIT, boosted SIT with pupicide pyriproxifen (BSIT), and/or auto dissemination technique (ADT). Additionally, the rate of rolling out sterile male mosquitoes and competitiveness threshold were identified.	DM	n/a	B	Boosting decreased the thresholds in sterile male release rate and fuelled the mosquito’s destabilisation. There was no bifurcation in the ADT sub-model. Also, BSIT could avert over 95% of the overall rollout to mitigate dengue burden than SIT, suggesting that BSIT is effective in the control management of *Aedes* mosquitoes.
22	Qu et al. [44]	2018	Developed a two-sex mosquito model to describe the potential effectiveness of *Wolbachia* transmission for controlling mosquito-borne diseases. This model accounts for the *Wolbachia* transmission dynamics and incorporates the aquatic stage and various pregnant stages of adult female mosquitoes and heterosexual transmission. The R0 was computed. A threshold effect, which is driven by a backward bifurcation with three coexisting equilibria, is identified. The sensitivity analysis of the model parameters and the effectiveness of different migration strategies were investigated.	DM	n/a	B	It was shown that if R0 is less than one, the endemic equilibrium can still be stable via the backward bifurcation effect. Furthermore, there is a threshold condition for which a proportion of mosquitoes must exceed for *Wolbachia* establishment to occur in wild-type mosquitoes. In addition, the best way to establish *Wolbachia* infection in mosquitoes is to decrease the wild-type mosquito population either by insecticide spraying or mosquito traps and then introduce male and pregnant female mosquitoes infected with *Wolbachia* infections.
23	Robert et al. [58]	2013	A reduce and replace (RandR) strategic model, which numerically accounts for the release of insects (dengue vector *Aedes aegypti* mosquitoes) possessing the anti-pathogenic and female-killing trait, was proposed. In other words, this model described the strategic release of *Aedes aegypti* mosquito carrying RandR strain to suppress mosquito-borne diseases, such as dengue.	DM	n/a	B	Following the modelling results, it was shown that continuous release of RandR may temporarily reduce the density of the *Aedes* mosquito population, and this reduction may be long-lasting in the absence of fitness cost being related to the anti-pathogenic gene. Also, the swift RandR strain releases have a long-term reduction of vector densities compared to only female-killing rollout. Furthermore, the degree of reduction in overall mosquito densities depends on female inclusion in the rollout strategy, the release duration and release proportion.
24	Salami et al. [59]	2020	A deterministic model was adopted to portray the transmission dynamics of dengue in the *Aedes aegypti* mosquito population. This model accounts for the influence of seasonal fluctuating temperatures by integrating empirical and idealistic parameter tools. The epidemic dynamics of the seasonality influence were investigated following an imported case via the arrival of an infectious person. A sensitivity analysis was also performed on the interested quantities: peak time, epidemic peak size, and final epidemic size.	DM	Funchal, Madeira Island	E	The model results showed that the autumn and summer seasons could fuel dengue transmission, with the arrival date of an infectious person greatly affecting the time and peak size distribution of the dengue epidemic. Interestingly, late-summer infectious individual arrivals could generate large epidemics within a short time amplitude. It was also revealed that seasonality affects the epidemic dynamics. This suggests that large epidemics with a short time amplitude could be produced with starting warm temperatures and vice versa. The sensitivity analysis showed that the interested quantities were most sensitive to changes in the arrival date, seasonal temperature, mortality and transmission rates and mosquito population.
25	Senapati et al. [60]	2019	A general multi-patch dengue model was formulated to describe the Spatio-temporal transmission dynamics of dengue disease and the effectiveness of various adult mosquito controls (i.e., efficacy and environmental persistence) to reduce the dengue burden. This model was fitted to monthly data of dengue cases in five regions of Kolkata, India, for a period of two years (from 2014 to 2015).	SM	India: Kolkata	E	The results showed that control strategies with higher environmental persistence are more effective compared with the strategies with low environmental persistence. Also, the effectiveness of adult control strategies is greatly influenced by the spatial coupling (connectedness) between the regions. Amongst the three control strategies considered—ultra-low-volume (ULV) spray of insecticides; insecticide treatment of surfaces and materials; and use of lethal ovitraps—the most effective in reducing dengue cases is the treatment of surfaces and materials, while the least effective is ULV.
26	Strugarek et al. [34]	2019	Derived a minimalistic mathematical model incorporating the sterile insect technique (SIT) and incompatible insect technique (IIT) to eliminate the *Aedes* mosquito population. Unlike other previous models, the model considered in this study is bistable as it accommodates mosquito population elimination and survival. Different types of releases, which are constant, periodic, or impulsive releases, were considered as the necessary conditions for elimination were shown. Estimation of the parameters using an *Aedes polynesiensis* population study and both sufficient and minimal treatment times were performed, and both analytical and numerical results were analysed.	DM	n/a	B	The results showed that the mating competitiveness of the SIT control strategy needs to be close to one for effectiveness. If this is not the case, there may be limited efficacy if there are too few numbers of wild-type mosquitoes. Also, the mating parameter in the model is very important in the duration of controlling vectors via the SIT method, and it is suggested that entomologists focus more on the probability of mating between a male and a female mosquito with respect to the size of their habitat in their prospective experiments.
27	Tang et al. [61]	2016	Developed a mathematical model to imitate the impulsive vector control program and continuous treatment of patients and isolation in the Guangdong Province of China during the 2014 dengue outbreak. This vector program has occurred every week (specifically on Friday afternoons) since its inception. The accumulated dengue infection data were fitted using the parameterized model to perform a retrospective analysis. This analysis was used to estimate the basic and control R0 and the mosquito-killing ratios.	DM	China: Guangdong	C	The results showed the estimation of both basic and control R0 to be 1.7425 and 0.1709, respectively, suggesting a highly effective control of the dengue outbreak during the intervention program. It was also observed that when a Friday was skipped during the integrated program, this would not increase the control R0 to one; rather, it would increase the number of accumulated infections at the end of the disease outbreak. In all, a rapid and regular impulsive vector control implementation leads to an effective decrease in the control R0, which in turn significantly reduces new infections.
28	Thome et al. [62]	2010	Presented a mathematical model that captured the introduction of sterile male mosquitoes, besides the use of chemicals (insecticides), to biologically control the mosquito population. The optimal control strategy was used to search for the minimal effort required to decrease female mosquitoes that are productive by considering the cost of sterile male mosquito production, the cost of delivery to experimental sites, together with the social cost, and the cost of chemical application, such as insecticide.	DM	n/a	B	The model results showed that the social cost should be considered in controlling mosquito vectors as its exception when reducing the cost of other control strategies could result in unsuitable strategies. Furthermore, at the initial stage of the control strategy, high chemical insecticide application was required and then gradually decreased with time. Unless the social cost was multiplied by a hundred, the sterile male mosquito release should follow a bell-like curve with an increase and decrease at both ends together with a moderately flat middle.
29	Wijaya et al. [63]	2014	Presented an optimal control model, which described the dynamics of mosquito reduction management using chemicals, such as temephos, and conducted fumigation in dengue-endemic regions where mosquitoes are prevalent. The basic R0 was computed, and equilibrium stabilities were analysed.	DM	n/a	C	The results showed that if R0 is less than 1, the disease-free equilibrium (DFE) existed and was locally asymptotically stable, while the coexistence equilibrium (CE) did not exist. On the other hand, if R0 was greater than 1, the DFE was unstable, but the CE existed and was globally asymptotically stable in a positive region. Also, the best mosquito control strategies obtained from the optimal control analysis were obtained if the number of mosquitoes was small at the initial stages of control and additionally combined the use of temephos and fumigating activities.
30	Winskill et al. [64]	2014	Designed a compartmental model that accounted for the release dynamics of adult and pupal mosquitoes carrying RIDL. This model was used to fit an experimental data, which described the large-scale pupal mark release/recapture phenomena to determine pupal release dynamics. The simulation of pulsed releases of adult, pupae, or the combination of both was shown. Various release mechanisms of mosquito-carrying RIDL to sustain a long-lasting decrease in the wild-type mosquito population are investigated.	DM	n/a	B	For regular recurring releases, model simulations showed that releasing only adult-carrying RIDL mosquitoes performs better compared with the other releases: pupae only and combined adult-pupae releases, and vice versa for less recurring releases. The relative efficacy of releasing pupae is affected by the pupal emergence rate from the release apparatus. For a sustained, long-lasting reduction of wild mosquitoes in the presence of low recurrence, the combined adult–pupae mosquito release is more effective than the pupae-only or adult-only releases.
31	Zhang and Lui [35]	2020	Developed a mathematical model to investigate the *Wolbachia* transmission dynamics in *Aedes-aegypti* mosquitoes as a means of suppressing the spread of dengue. This model considered only female mosquitoes as they give infectious bites or obtain protein via bites to maturate their eggs. Equal numbers of male and female mosquitoes were assumed. Sensitivity and optimal control analysis were performed on model parameters.	DM	n/a	B	The model analysis revealed that without release, the model is bistable. This indicates that only one interior steady state is stable whenever it exists. Optimal control theory showed that halting a release after a continuous release for two years would allow the *Wolbachia*-only equilibrium to be locally asymptotically stable with time, suggesting the invasion of *Wolbachia* in all the mosquitoes and then resulting in the prevention of the spread of dengue viral infection.
32	Zhang et al. [65]	2015	Proposed a model that described the spread and invasion of *Wolbachia* infections accounting for the effects of CI and fitness effects. This model explored whether augmentation could inhibit the transmission of dengue in the field and also considered the question of why some rollout strategies were unsuccessful and what caused this failure in establishing population replacement.	DM	n/a	B	The stability analysis showed that some phenomena may have contributed to the failure of the *Wolbachia* invasion in wild mosquitoes. Such attractors include backward bifurcation and augmentation mechanisms, such as frequency, quantity, and timing. In all, the modelling result revealed that the successful establishment of *Wolbachia* infection via replacing the wild mosquitoes with *Wolbachia*-infected mosquitoes would depend on the type of *Wolbachia* strains selected for deployment and appropriate augmentation techniques.

**Table 2:** Description of the quality assessment of the included studies adopting the tool Assessment for Modelling Studies (ASM). The values in the table below represent the AMS criteria 0 → absent, 1 → partially present, 2 → fully present.

S/No.	Author	Year	Aims and Objectives/Abstract	Intervention Comparators	Outcome Measures Defined	Model Structure and Flowchart	Modelling Methods	Parameters Specified	Assumptions Explicit and Justified	Quality of Data and Uncertainty and/or Sensitivity Analyses	Model Validation	Presentation of Results	Interpretation and Discussion of Results	Conflicts of or Competing Interest Declared	Final Point	Final Score (%)	Rating
1	Abad-Franch et al. [46]	2017	2	2	2	1	2	2	2	0	2	2	2	2	21	87.50	Very High
2	Agusto and Khan [37]	2018	2	1	2	2	2	2	2	2	2	2	2	0	21	87.50	Very High
3	Alphey et al. [38]	2011	2	1	2	2	2	2	2	2	1	2	2	2	22	91.67	Very High
4	Andraud et al. [39]	2013	2	2	2	1	2	2	2	0	2	2	2	0	19	79.17	High
5	Barmak et al. [47]	2014	2	2	1	1	2	2	1	1	2	2	2	2	20	83.33	Very High
6	Bliman et al. [32]	2019	2	1	1	1	2	2	2	0	1	2	2	2	18	75.00	High
7	Buonomo and Della Marca [48]	2018	2	2	2	1	2	2	2	0	1	2	2	0	18	75.00	High
8	Cai et al. [40]	2018	2	1	1	1	2	2	2	0	1	2	2	0	16	66.67	High
9	Campo-Duarte et al. [41]	2018	2	2	2	1	2	2	2	2	1	2	2	0	20	83.33	Very High
10	Chavez et al. [49]	2017	2	1	2	1	2	2	2	0	1	2	2	0	17	70.83	High
11	Hancock et al. [50]	2016	2	1	2	1	2	2	2	1	2	2	2	2	21	87.50	Very High
12	He et al. [51]	2017	2	1	1	1	2	2	2	0	1	2	2	2	18	75.00	High
13	Hughes and Britton [52]	2013	2	1	2	2	2	2	2	1	1	2	2	0	19	79.17	High
14	Li and Liu [53]	2018	2	2	1	1	2	2	2	1	1	2	2	0	18	75.00	High
15	Luz et al. [54]	2011	2	2	2	2	2	2	1	2	1	2	2	2	22	91.67	Very High
16	Marini et al. [33]	2019	2	1	1	2	2	2	2	2	2	2	2	2	22	91.67	Very High
17	Mishra et al. [55]	2018	2	1	1	2	2	2	2	0	1	2	2	0	17	70.83	High
18	Ndii et al. [42]	2016	2	2	1	2	2	2	2	2	1	2	2	0	20	83.33	Very High
19	Oki et al. [56]	2011	2	2	1	1	2	2	2	0	1	2	2	2	19	79.17	High
20	O’Reilly et al. [43]	2019	2	2	1	2	2	1	2	2	2	2	2	2	22	91.67	Very High
21	Pleydell and Bouyer [57]	2019	2	1	1	1	2	1	2	2	1	2	2	2	19	79.17	High
22	Qu et al. [44]	2018	2	2	1	2	2	2	2	2	1	2	2	0	20	83.33	Very High
23	Robert et al. [58]	2013	2	2	1	1	2	2	2	0	1	2	2	2	19	79.17	High
24	Salami et al. [59]	2020	2	2	1	2	2	2	2	2	2	2	2	0	21	87.50	High
25	Senapati et al. [60]	2019	2	2	1	1	2	2	2	0	2	2	2	0	18	75.00	High
26	Strugarek et al. [34]	2019	2	1	1	1	2	2	2	0	1	2	2	0	16	66.67	High
27	Tang et al. [61]	2016	2	1	1	1	2	2	2	2	1	2	2	0	18	75.00	High
28	Thome et al. [62]	2010	2	2	1	1	2	2	2	0	1	2	2	0	17	70.83	High
29	Wijaya et al. [63]	2014	2	2	1	1	2	2	2	1	1	2	2	0	18	75.00	High
30	Winskill et al. [64]	2014	2	2	1	1	2	2	2	0	2	2	2	2	20	83.33	Very High
31	Zhang and Lui [35]	2020	2	1	1	2	2	2	2	2	1	2	2	2	21	87.50	Very High
32	Zhang et al. [65]	2015	2	2	1	1	2	2	2	1	1	2	2	0	18	75.00	High

**Table 3:** Table showing the distribution of study quality with respect to the vector control types.

	Study Quality	Very High	High	Medium	Low	Total
Vector Control Types						
**Chemical**		5 (50%)	5 (50%)	0	0	10
**Biological**		8 (42%)	11 (58%)	0	0	19
**Environmental**		0 (0%)	3 (100%)	0	0	3

## Supplementary Materials

The following supporting information can be downloaded at: https://www.mdpi.com/article/10.3390/v15010254/s1. Additional File S1: Search Strategies for Systematic Review.
