# Supplementary material for: A Systematic Review of Mathematical Models of Dengue Transmission and Vector Control: 2010–2020
Source: Viruses. 2023 Jan 16;15(1):254. doi: 10.3390/v15010254 (PMC9862433; doi:10.3390/v15010254)
Supplement: Supplementary file 1 [file viruses-15-00254-s001.zip › viruses-2154764-supplementary.pdf]

SEARCH STRATEGIES FOR SYSTEMATIC REVIEW

A systematic review of dengue transmission models: vector control approach.

MEDLINE: (1069 articles)

1. *arbovirus infections/ or exp dengue
2. *flavivirus/ or *dengue virus/
3. 1 or 2
4. exp Models, Biological
5. exp disease outbreaks/ or exp disease transmission, infectious
6. exp *Disease Models, Animal/
7. 4 or 5 or 6
8. exp Culicidae/
9. exp Disease Vectors/
10. exp Mosquito Nets/
11. exp Insect Control/
12. *arthropods/ or insecta/
13. 8 or 9 or 10 or 11 or 12
14. exp communicable disease control/ or infection control/
15. exp population dynamics/ or population control/
16. *Infection Control/
17. *Disease Eradication/
18. 14 or 15 or 16 or 17
19. 13 and 18
20. 3 and 7
21. 19 and 20

exp -> a search term was “exploded” in the MeSH (Medical Subject Headings) from MEDLINE vocabulary to also capture all narrower terms associated with the broader concept.

WEB OF SCIENCE: (643 articles)

TOPIC: (dengue OR arbovirus* OR flavivirus*) *AND*

TOPIC: (transmi* model* OR infect* model*) *AND*

TOPIC: (vector* OR mosquito* OR insect* OR Aedes OR arthropod*) *AND*

TOPIC: (control* strateg* OR control* techni* OR control* approach* OR control* method*)

Timespan: All years.

Indexes: *SCI-EXPANDED, SSCI, A&HCI, CPCI-S, CPCI-SSH, ESCI, CCR-EXPANDED, IC*

SCOPUS: (446 articles)
((TITLE-ABS-KEY (dengue OR arbovirus* OR flavivirus*)) AND (TITLE-ABS-KEY (transmi* AND model* OR infect* AND model*))) AND ((TITLE-ABS-KEY (vector* OR mosquito* OR insect* OR aedes OR arthropod*)) AND (TITLE-ABS-KEY (control* AND strateg* OR control* AND techni* OR control* AND approach* OR control* AND method*)))
